# Ultrafast fMRI Detects Age‐Related Changes in Harmonics of Cardiac Pulsations in the Brain at 7 T

**DOI:** 10.1002/mrm.70429

**Published:** 2026-05-10

**Authors:** Charles Marchini, Dema Abdelkarim, Yuhui Chai, Monica Fabiani, Gabriele Gratton, Bradley P. Sutton

**Affiliations:** ^1^ Beckman Institute for Advanced Science and Technology, University of Illinois at Urbana‐Champaign Urbana Illinois USA; ^2^ Grainger College of Engineering, Bioengineering Department University of Illinois at Urbana‐Champaign Urbana Illinois USA; ^3^ Carle Illinois Advanced Imaging Center Carle Hospital and University of Illinois at Urbana‐Champaign Urbana Illinois USA; ^4^ Neuroscience Program, University of Illinois at Urbana‐Champaign Urbana Illinois USA; ^5^ Department of Psychology University of Illinois at Urbana‐Champaign Champaign Illinois USA; ^6^ Carle Illinois College of Medicine, University of Illinois at Urbana‐Champaign Urbana Illinois USA

**Keywords:** 7 Tesla, aging, arterial stiffening, BOLD fMRI, cerebrovascular pulsatility, partial separability

## Abstract

**Purpose:**

Aging impacts pulsatility of blood vessels in the brain, reflecting vascular health. Resolving characteristics of cardiac pulsatility requires faster acquisitions than traditional fMRI. An ultrafast fMRI method was developed and applied to sample several harmonics of cardiac pulsations at 7 T.

**Methods:**

Leveraging partial separability, an ultrafast fMRI method (TR = 65 ms) was applied at 7 T with full brain coverage at 2 mm isotropic resolution and concurrent pulse plethysmographic (PPG) recordings in 28 healthy adults, 22 of whom had acceptable PPG and MRI data. The reliability of the pulsation was assessed for each voxel through even/odd splitting of heartbeats. The PPG was used to retrospectively temporally align signals across heartbeats, and analysis was performed on amplitudes of harmonic frequencies to explore age‐ and sex‐related differences. Reliable measures of four harmonics of the heartbeat were characterized along with the ratios of the harmonics to the primary harmonic.

**Results:**

High reliability of the pulsation was found near major arteries, tested by Spearman correlation between the first harmonic and a vessel atlas (*p* < 0.0001). Consistent with previous literature, older adults exhibited higher harmonic amplitudes with the strongest result in the second harmonic in gray matter with FDR‐corrected *q* = 0.020. In addition, males had a higher third‐to‐first harmonic ratio.

**Conclusion:**

Ultrafast fMRI can reliably resolve several harmonics of cardiac pulsations in the brain in and around blood vessels. This may provide a way to characterize vascular health throughout the brain during aging.

## 1 Introduction

Age‐related changes in cognitive and brain function can lead to the development of mild cognitive impairment and dementia. Risk factors for these outcomes largely overlap those for cardiovascular and cerebrovascular disease. Epidemiologic studies demonstrate that age‐related vascular problems are associated with poor cognitive outcomes in older adults [1, 2]. Age‐related differences in brain tissue atrophy have been shown to reflect individual differences in cardiorespiratory fitness [3, 4, 5].

Aging is typically associated with arterial function changes, such as cerebral arteries decreasing in elasticity (arterial stiffening). Arterial stiffening leads to faster pulse wave velocity [6], which modifies the shape of the arterial pulse, due to increased temporal overlap between the forward wave generated by the heart pulsation and the corresponding backward wave generated by peripheral resistance at muscular arteries and arterioles [7]. This leads to larger and sharper pulse waves in older adults.

It should be noted, however, that although generated within the arteries, the cardiac pulsatile motion can propagate through tissue and also be observed in brain parenchyma. This motion occurs throughout the brain, and while the term “pulsatility” can be used in different ways, here we refer to pulsatility as the degree to which a signal occurs at the frequency band of cardiac pulsation. Cerebral pulsatility has been of increasing interest for relevance in neurological aging, neurological disease, and resting‐state fMRI studies. Cerebrovascular pulsatility studies have found that age‐related arterial stiffening may produce changes in dampening effects on pulsatile flow into capillaries [8, 9]. In addition to providing an understanding of vascular wall changes with age, pulsation around blood vessels is also assumed to have a critical role in glymphatic flow and in the clearance of amyloid‐β associated with Alzheimer disease [10]. For these reasons, major strides have been made in the development of methods to estimate, directly measure, and parameterize pulsatile fluid dynamics in the brain [11].

Vascular aging has been measured in the brain using diffuse optical methods that characterize cerebral pulsation waveforms using a high temporal resolution and high sensitivity to oxy/deoxy hemoglobin [12, 13]. Previous studies have shown that local changes in arterial elasticity are associated with brain volume changes in the same regions [14]. However, optical imaging approaches have limitations when it comes to depth of penetration, which in adults prevents studies of changes in cerebrovascular reactivity in deeper cortices and subcortical structures. Translation of pulsatile imaging to MRI would enable analysis of deeper cortical regions such as the hippocampus.

BOLD fMRI offers superior spatial resolution and whole‐brain imaging to examine vascular elasticity and reactivity. BOLD has also been used with CO_2_ inhalation or breath‐holding tasks to measure cerebrovascular reactivity [15, 16, 17]. However, fMRI studies with a typical repetition time (TR) of 1 s lack the temporal resolution required to resolve the detailed cardiac pulse waveform to examine vascular elasticity. At typical resting‐state fMRI sampling rates between 0.5 and 1 Hz, a typical resting heart rate would exceed the Nyquist limit even for the primary harmonic [18], and the pulse signal would be aliased at other frequencies. However, characterization of the waveforms can be achieved with the fMRI data after it has been retrospectively cardiac‐gated to align with the cardiac phase [19, 20, 21].

Previous studies modified fMRI protocols with shorter TR to achieve better characterization of cardiac pulsations in the brain, motivated by both the methodological benefits of this characterization (more effective removal of physiological noise from resting‐state fMRI data) as well as the ability to analyze the pulsations to understand cerebrovascular dynamics. Although physiological noise is often a nuisance signal in resting‐state fMRI, several studies targeted the physiological variations in BOLD fMRI as a signal with useful information [19, 22, 23, 24, 25, 26, 27]. For example, one study used a 250 ms TR with EPI to cover a small volume (four 5‐mm slices) of the brain to measure the average amplitude around the heartbeat frequency in normal appearing white matter (WM) and found pulsatility differences occur between young adults, old adults, and old adults with small vessel disease [24]. To cover a significant volume at the same TR, simultaneous multislice imaging can be used, which involves simultaneously sampling multiple slices to increase coverage within a TR [19].

In addition to directly imaging the pulsatile waveforms with fast BOLD fMRI sequences, the pulse plethysmograph signal (PPG) measured simultaneously with the resting state fMRI scan can be used to determine heart rate and cardiac phase for retrospective realignment [19, 20, 26, 28, 29]. These methods require merging data over many heartbeats to obtain sufficient samples across the temporal profile of the cardiac waveform. With this approach, previous literature has shown a decrease in BOLD signal in the arteries during systole and localization of the pulse waveform to the major arteries and CSF [19, 20, 26, 30]. Several studies with a retrospective alignment measured pulsatility by fitting 7‐term Fourier series to the cardiac‐phase‐aligned fMRI data (retrospectively aligned using PPG signal) and measuring pulsatility as the fit of the curve [28, 29]. Some studies use the amplitude of the retrospectively aligned curve as a pulsatility measure, which can be done after computing percent signal changes [19], by normalizing to the standard deviation across time for the voxel [20], or by normalizing to a baseline signal with low pulsatility [21]. When the cardiac frequency is within Nyquist limits, pulsatility can be defined as average amplitude within a given interval of the heart rate frequency [24]. Using a 0.02 Hz interval, WM hyperintensities associated with small vessel disease exhibited lower pulsatility than normal WM; however, older adults showed higher pulsatility in normal WM than younger adults [24]. Both extremes of pulsatility can indicate vascular dysfunction. A U‐shaped association was found between the pulsatility index (measured by ultrasound) and trail‐making test, where both low and high pulsatility were correlated with increases in time to complete the test [31].

Other novel MRI‐based approaches have been able to achieve high temporal resolution. A 3D fast method called magnetic resonance encephalography (MREG) uses spatial localization from large receiver array coils to achieve faster functional imaging [30]. MREG is able to achieve the Nyquist sampling of the heartbeat, but suffers from limited spatial resolution [30]. It has been used to detect heart rate harmonic power and link differences to Alzheimer's disease [32]. Harmonics are a relevant feature of aortic blood pressure waveforms and have been shown to be related to the biomechanics and stiffness of vessels [33]. In this work, we develop a sequence to achieve whole brain, high spatiotemporal resolution imaging to properly resolve cerebral pulse signals. We use a partial separability (PS) fMRI acquisition and reconstruction [34, 35, 36]. The PS model has previously been applied to dynamic imaging to acquire high‐speed images of speech [37, 38]. The acquisition and reconstruction were adapted for fMRI. We demonstrate this partial separability fMRI (PS‐fMRI) sequence allows for pulsatility detection robustly at the first four cardiac harmonics. We characterize reliability by a voxel‐by‐voxel measure based on splitting even/odd heartbeats, similar to previous work [19]. With full‐brain coverage, we compare pulsatility in WM, gray matter (GM), cerebrospinal fluid (CSF), and the circle of Willis (CoW) within the reliable pulse regions. To demonstrate the validity of the pulsatility signal, we replicated previous findings of increased pulsatility in older adults relative to younger adults [9, 21, 24].

Sex is often included as a variable in examining vascular changes with age due to sex‐related differences in neurovascular aging [39]. Pulsatility and pulsatile damping (decrease in pulsatility index as wave travels) have been shown to have sex‐related differences with phase‐contrast MRI [40]. Therefore, we included sex as a covariate in our analysis and examined relationships between sex and harmonics of pulsatility.

In summary, the primary aim of the study is to demonstrate the use of our ultrafast fMRI sequence to compare harmonic differences across age and sex throughout the brain. Consistent with previous literature, we expect differences between age groups due to vascular system differences, as aging brings higher pulsatility. Additionally, we anticipate potential differences across sex due to previous reports indicating sex differences in the cardiovascular system [41].

## 2 Methods

### 2.1 Participants

All procedures in this study were approved by the Institutional Review Board of the University of Illinois at Urbana‐Champaign. Twenty‐eight volunteers were recruited to this study from a pool of participants that had previously taken part in studies in the laboratory. Participants were asked if their health status had changed from the last appointment and were screened for MRI contraindications. They were instructed to refrain from exercise, caffeine, or use of illicit or vasodilatory drugs for 24 h prior to their appointment. Upon arrival for their scan, volunteers gave informed consent to participate and were screened for cognitive impairment using the Montreal Cognitive Assessment [42]. Of the 28 participants' data collected, some were dropped prior to the analysis phase: 2 due to missing physiological files, 2 due to missing anatomical data, and 2 due to corrupted PPG, leaving 22 subjects before outlier removal, which removed 3 additional participants as explained in Section 2.5.2. Demographics for the subjects prior to outlier removal are shown in Table 1.

**TABLE 1:** Demographic information for sample before outlier removal.

	All	Older	Younger	*p*	Female	Male	*p*
*N*	22	14	8	—	10	12	—
Age *M* (SD)	53.0 (17.6)	65.6 (5.0)	31.0 (3.0)	< 0.001	50.3 (17.2)	56.3 (18.2)	0.445
MoCA *M* (SD)	28.1 (1.6)	27.9 (1.5)	28.6 (1.9)	0.404	27.5 (1.3)	28.7 (1.8)	0.102

### 2.2 Scanning Procedure

Anatomical, PS‐fMRI with concurrent PPG, and angiogram scans were performed using a Siemens Magnetom Terra 7 T scanner at the Carle Illinois Advanced Imaging Center. Participants were safety screened an additional time by an MRI scan technician prior to scanning. For physiological data collection, participants were outfitted with the MRI scanner provided respiration belt across their chest and pulse oximeter on one finger, interfaced through the Siemens wireless hardware. The scan session lasted approximately 1 h, with the fast fMRI scan lasting 10 min 24 s, acquired in the first half of the scan session. Participants were asked to stay awake and stare at a white cross on a black background for the duration of the PS‐fMRI scan.

### 2.3 PS‐fMRI Sequence and Reconstruction

The PS‐fMRI acquisition was 3D with full brain coverage, 2 mm isotropic resolution, a TR = 65 ms, and TE = 25 ms. We used 7 T as the contribution of physiological signal relative to thermal noise in the fMRI timeseries increases with field strength [43].

**FIGURE 1:**
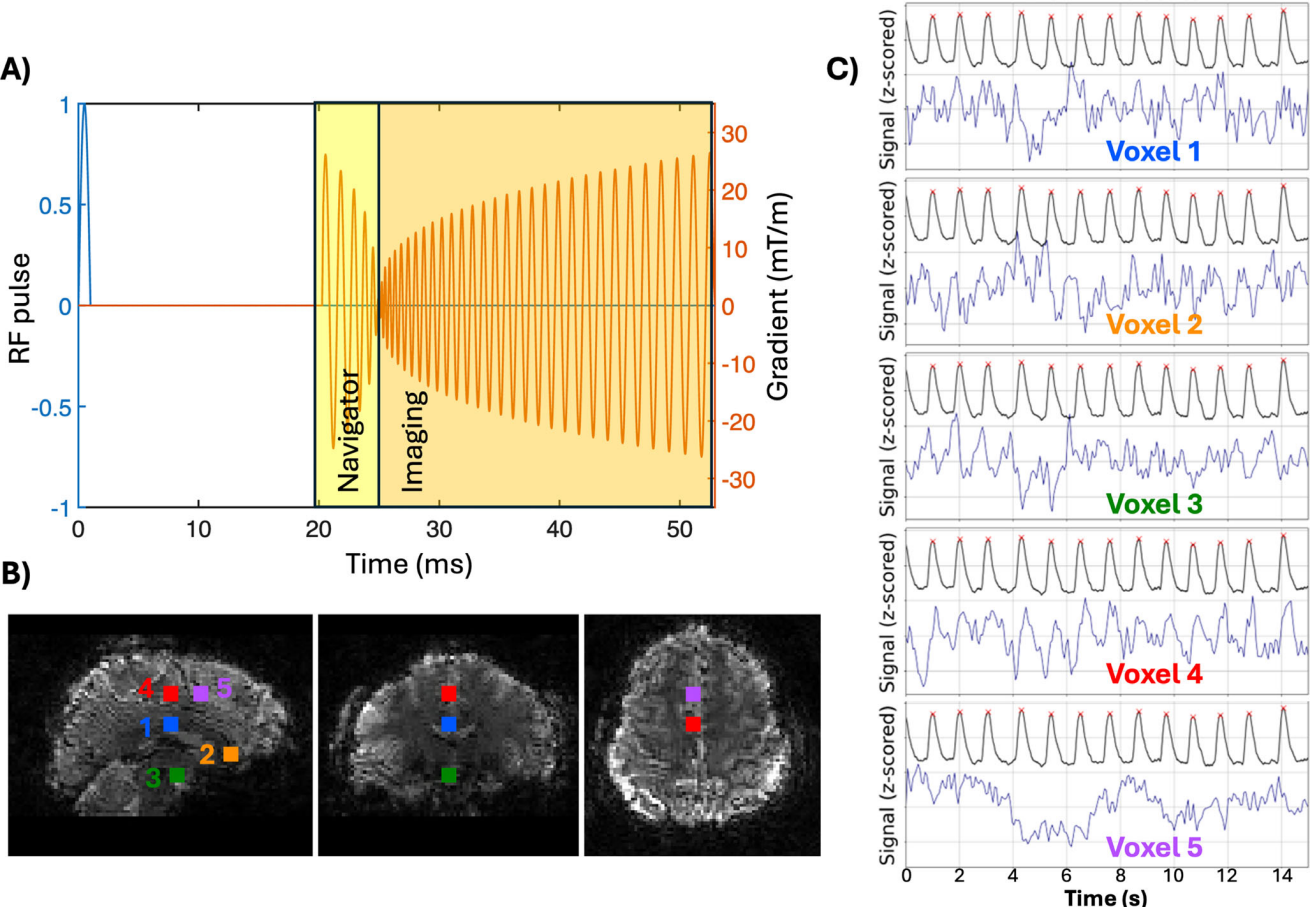
PS‐fMRI sequence and imaging results. (A) The PS‐fMRI sequence has an echo time of 25 ms, with a spiral‐in temporal navigator that is repeated every TR and a spiral‐out imaging data collection that samples a different shot and kz line each TR. (B) Resulting images from a single time point of the reconstructed PS‐fMRI acquisition showing five example voxel locations for examining the time series. (C) A 15 s time series of the five voxel locations in B. The figures show the PPG signal in black, with offset added for visualization, with PPG peak marked in red and the pixel time course shown in blue.

In order to achieve full‐brain coverage and high spatiotemporal imaging, the PS‐fMRI sequence leveraged the PS framework [34], where a spatiotemporal image is represented by a sum of the product of a small number of spatial and temporal basis functions:$\;m\left(t,r\right)=\sum_{l=1}^{L}\varphi_{l}{\left(t\right)}^{*}c_{l}\left(r\right)=\varphi {\left(t\right)}^{H}c\left(r\right)$, where m(t,r) is the spatiotemporal fMRI image over time, t, and space, r; L is the number of basis functions chosen; $\varphi_{l}\left(t\right)$ are the temporal basis functions in vector φ(t); and $c_{l}\left(r\right)$ are the spatial basis functions in vector c(r). For the PS‐fMRI acquisition, see Figure 1, a specially designed pulse sequence is used with an interleaved spiral‐in temporal navigator and a stack‐of‐spiral out imaging data acquisition, sharing a TE of 25 ms. For the temporal navigator, a small, repeated region of k‐space is acquired immediately prior to each imaging acquisition. The temporal basis was estimated by performing singular value decomposition of the temporal navigator data and retaining the top L right singular vectors. The imaging spatial acquisition samples all of k‐space in a multi‐shot, stack‐of‐spirals approach for the corresponding spatial maps. After estimation of the temporal basis, the spatial basis is estimated in a least squares estimation framework:

$$\hat{c}\left(r\right)=\arg\min_{c\left(r\right)}{\left\|\;s\left(t,k\right)-Ϝ\left(\varphi {\left(t\right)}^{H}c\left(r\right)\right)\right\|}_{2}$$

where s(t,k) is the sampled imaging data at the sparse k‐space locations for each time point and Ϝ is an operator that transforms the image model into k‐space and applies the sparse sampling operator. The PS framework enables the estimation of space and time with separate but interleaved acquisitions to create a high spatiotemporal image series [37, 38].

For the spiral‐in temporal navigator, a short (1 shot of a 12‐shot spiral design, only kz = 0, 4.65 ms readout) readout was used. The spiral‐out imaging acquisition had 2‐shot per kz‐plane with acceleration factor of 2 (4‐shots designed, 2 shots 180° rotated) readout with 24 cm field of view with matrix size of 120 and 60 kz‐planes yielding an isotropic resolution of 2 mm. Only one shot in one kz‐plane was acquired for imaging data per acquisition (coupled with a fixed temporal navigator) with sequential sampling of kz‐lines. The gradient waveform for a single TR is shown in Figure 1A. The flip angle was 20° and fat saturation was used with a chemical shift selective module [44]. In the 10‐min, 24‐s‐long acquisition, 9600 temporal image frames are acquired. The image reconstruction [37] proceeds as described above: choosing L=35 components, and estimating the associated spatial basis to provide a full framerate reconstruction of the timeseries. Figure 1C demonstrates the time series for one subject for five voxels whose location is shown in Figure 1B. In addition, a sensitivity map and field map estimation sequence was acquired just prior to the PS‐fMRI sequence to assist in reconstruction.

Due to residual coherency in sampling with a non‐random kz line order, some residual temporal spikes were present in the timeseries. To eliminate this spiking, the fBIRN small QC phantom (EZfMRI, Chicago, IL) was scanned with the same protocol demonstrating the same sharp noise spikes. Ignoring the first and last 250 points in the 9600‐point spectrum, the signal was thresholded, resulting in a mask of 160 frequency locations (out of 9600) that are removed from the spectrum of the temporal basis in each participant's data to eliminate the sharp frequency spikes. See Figure S1 for visual inspection of the filter's performance.

### 2.4 Data Processing

#### 2.4.1 Physiological Data

Physiological data output from the scanner was cleaned to remove header information and non‐measurement codings, then aligned to the beginning of the fMRI scan.

#### 2.4.2 Anatomical Data

Anatomical T1‐weighted images for registration were collected using a Magnetization Prepared Two Rapid Acquisition Gradient Echo (MP2RAGE) sequence [45] with scan parameters TR = 4.53 s, TE = 2.26 ms, inversion times = 750/2950 ms, flip angles = 4° and 5°, voxel size = 0.75 × 0.75 × 0.75 mm^3^. MP2RAGE images were denoised using the LayNii package [46] and brain extraction was performed using FSL BET [47]. Anatomical data were segmented by tissue type using FreeSurfer 5.3.0 [48] to obtain GM, WM, and CSF partial volume masks. The masks were made binary by thresholding at 0.5. Time‐of‐flight angiography was also employed to obtain maps of vasculature (TR = 8.3 ms, TE = 2.46 ms, flip angle = 20°, voxel size = 0.5 × 0.5 × 0.5 mm^3^).

#### 2.4.3 Functional Data

Registration of fMRI to MNI space was performed with advanced normalization tools (ANTs) in python [49]. The sensitivity map reference images were used as the PS‐fMRI reference as they were taken during the same scan and had the same dimensions as the fMRI scan. Data were linearly registered to anatomical and SyN transformed (affine then warp) to MNI space. The transformations were saved so they could be applied to results in the native fMRI space of each subject.

### 2.5 Analysis

#### 2.5.1 Pulse Reliability

For each subject, the first 300 repetitions were removed to reach steady state. The PS‐fMRI data were then broken up into 18 windows of 32.5 s (500 time points) with the last 300 time points unused. Each window was analyzed for pulse reliability, a voxel‐wise measure of the quality of the pulsatile fMRI signal, after band‐pass filtering from 0.1 to 5.5 Hz. Pulse reliability was measured by retrospective cardiac gating the time series into 30 bins according to the position of the time point in the cardiac phase, measured linearly from 0 to 1 for preceding peak in the PPG signal to the next peak, for even and odd heartbeats separately, then determining the coefficient of determination (*R* ^2^) between them, which is similar to the method described by Hermes et al. [19]. Pulse reliability measures the quality of the pulse signal within each voxel and each time window. We note that different temporal delays from the PPG occur due to different locations in the vascular tree. To minimize the impact of this on our results, we look at reliability of the pulse signal across a PPG‐indicated heartbeat and we look at signal energy around harmonics of the heartbeat frequency. Neither of these measures will be impacted by differential delays in different voxel locations.

The pulse reliability maps were averaged across temporal windows for each subject by first converting the *R* ^2^ to a z‐score using the Fisher z‐transform and then averaging across the windows, followed by using the inverse Fisher z‐transform to return to *R* ^2^ values for each voxel for each subject. Transformations described in 2.4.3 were applied to each *R* ^2^ map to convert them to MNI space and averaged across subjects, again using the Fisher z‐transform, to get a combined average *R* ^2^ in the MNI space. The group reliability map was thresholded at 0.4 to make a pulse reliability mask for restriction of further analysis to voxels with high pulse reliability. The same reliability mask was used across all participants.

#### 2.5.2 Pulsatility

Percent cardiac pulsatility was measured in a manner similar to Makedonov et al. [24]. Each voxel's unfiltered FFT magnitude was computed, normalized to the DC component, and the average magnitude within 0.15 Hz of the heart rate, as found from the PPG of the window using *findpeaks* in SciPy, was calculated and multiplied by 100 to compute percent cardiac pulsatility for that window. Harmonic ratios were defined as (% cardiac pulsatility of harmonic)/(% cardiac pulsatility of first harmonic). A CoW binary mask was created manually in MNI space as a sphere of voxels with a radius of 20 voxels in the CoW location. WM, GM, CSF, and CoW masks were transformed into the native fMRI space for each subject to compute the percent cardiac pulsatility within each region. The group pulse reliability mask was transformed from MNI to each subject's native fMRI space. Only voxels within the MNI brain mask and pulse reliability mask registered to each subject's native fMRI space were included in the analysis. Percent cardiac pulsatility was first averaged across windows for each subject, then averaged within each ROI according to each tissue type mask. Figure S2 shows MNI masks for WM, GM, CSF, and CoW to demonstrate what voxels remain after pulse reliability filtering when determining pulsatility metrics. The MNI masks were used when computing the Spearman correlations described in Section 2.6 to examine correlations between pulse reliability and pulsatility.

*Outlier windows detected within each subject*: Outlier time windows, defined as having percent cardiac pulsatility within any region (GM, WM, CSF, CoW) for any harmonic below the first quartile minus 3 times the IQR or above the third quartile plus 3 times the IQR, were removed before averaging. Across all subjects, a total of 24 time‐windows were removed. The greatest number of windows removed from one subject was 4.

*Outlier removal across subjects*: Outlier subjects were determined within young and old age groups separately. If a subject had an extreme value for any mask and harmonic, the subject was determined to be an outlier. An extreme value was a mean pulsatility (mean taken across non‐outlier windows) above the third quartile plus 3 times the interquartile range or below the first quartile minus 3 times the interquartile range. Subject outlier removal removed 1 young male, 1 young female, and 1 old female, leaving 19 total subjects (2 young males, 4 young females, 7 old males, and 6 old females) for statistical analysis.

#### 2.5.3 Correlations With Pulse

Prior to computing correlations between PPG and the fMRI signal for each voxel, PPG recordings were smoothed and resampled to the temporal resolution of the PS‐fMRI scan. FMRI time series were also linearly detrended via first order polynomial regression. MATLAB *corrcoef* was used to calculate the Pearson correlation coefficient between the time series of each voxel with the PPG.

### 2.6 Statistics

An ordinary least square test was used to assess differences in pulsatility between younger and older adults, using sex as a covariate and treating age group as a categorical variable. To correct for multiple comparisons within each harmonic metric, the false discovery rate method [50] was applied using multiple tests from *statsmodels.stats.multitest* in *python* with the “fdr_bh” method at alpha = 0.05 [51].

To determine whether pulse reliability and pulsatility were localized to vessels, Spearman correlation coefficients were computed between the pulse reliability map and vessels from a previously published atlas [52]. The vessel map was resampled to the 1 mm isotropic MNI template and clipped from 0 to 100 (probability 0 to 1). Then, after applying an MNI brain mask, a pulse reliability mask of *R* ^2^ > 0.05, and a vessel (large artery) map mask of probability > 0.05, the rank order Spearman correlation between the pulse reliability map and the vessel probability mask [52] was computed within the binary WM mask and within the binary GM mask. In addition, we computed the correlation between the percent cardiac pulsatility at each harmonic with the pulse reliability within the MNI brain mask. We also calculated the correlation between the percent cardiac pulsatility at the first harmonic and the vessel probability map in the union of the binary WM and GM masks.

## 3 Results

### 3.1 Pulse Reliability

**FIGURE 2:**
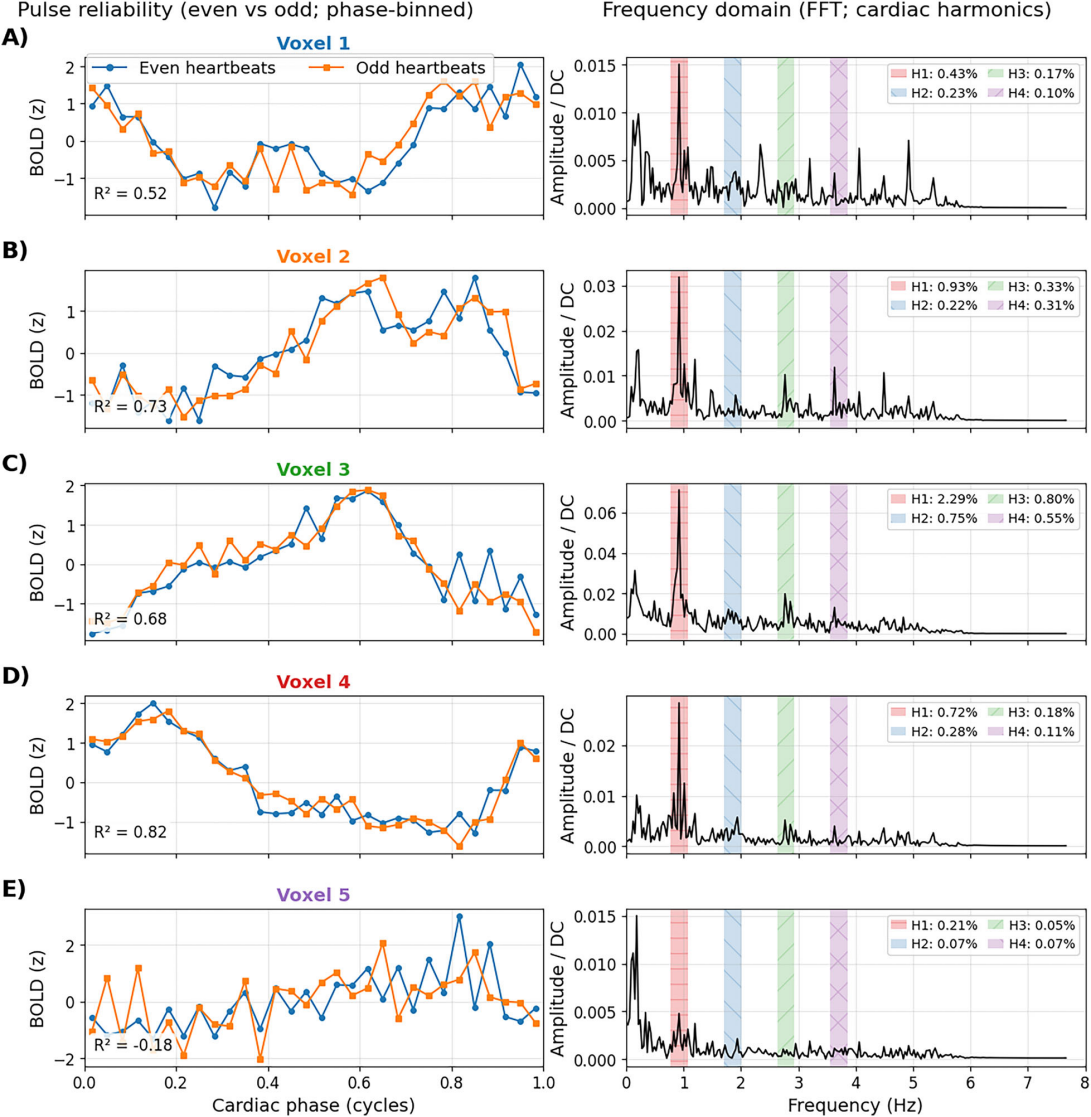
Individual voxels from Figure 1 from one subject for one 32.5 s time window. (A) Voxel 1, realigned pulse with reliability (top left), and frequency spectra (top right); (B–E) realigned pulse for Voxels 2–5 (left) and frequency spectra for Voxels 2–5 (right). Voxels 1–4 have high pulse reliability, indicating a quality heartbeat signal. Voxel 5 has low pulse reliability.

An example of pulse reliability measurements for five voxels is shown for one subject and for one time window (32.5 s) in Figure 2. The pulse‐realigned fMRI signal curves show that voxels corresponding to CSF have a signal with a peak near the PPG systole (cardiac phase 0 and 1) [19], with a CSF voxel shown in Figure 2A (Voxel 1) and Figure 2D (Voxel 4). Large arteries have a dip at systole [19], which occurs close to the PPG systole, shown in Figure 2B,C (Voxels 2 and 3). Figure 2E shows a voxel with low pulse reliability.

**FIGURE 3:**
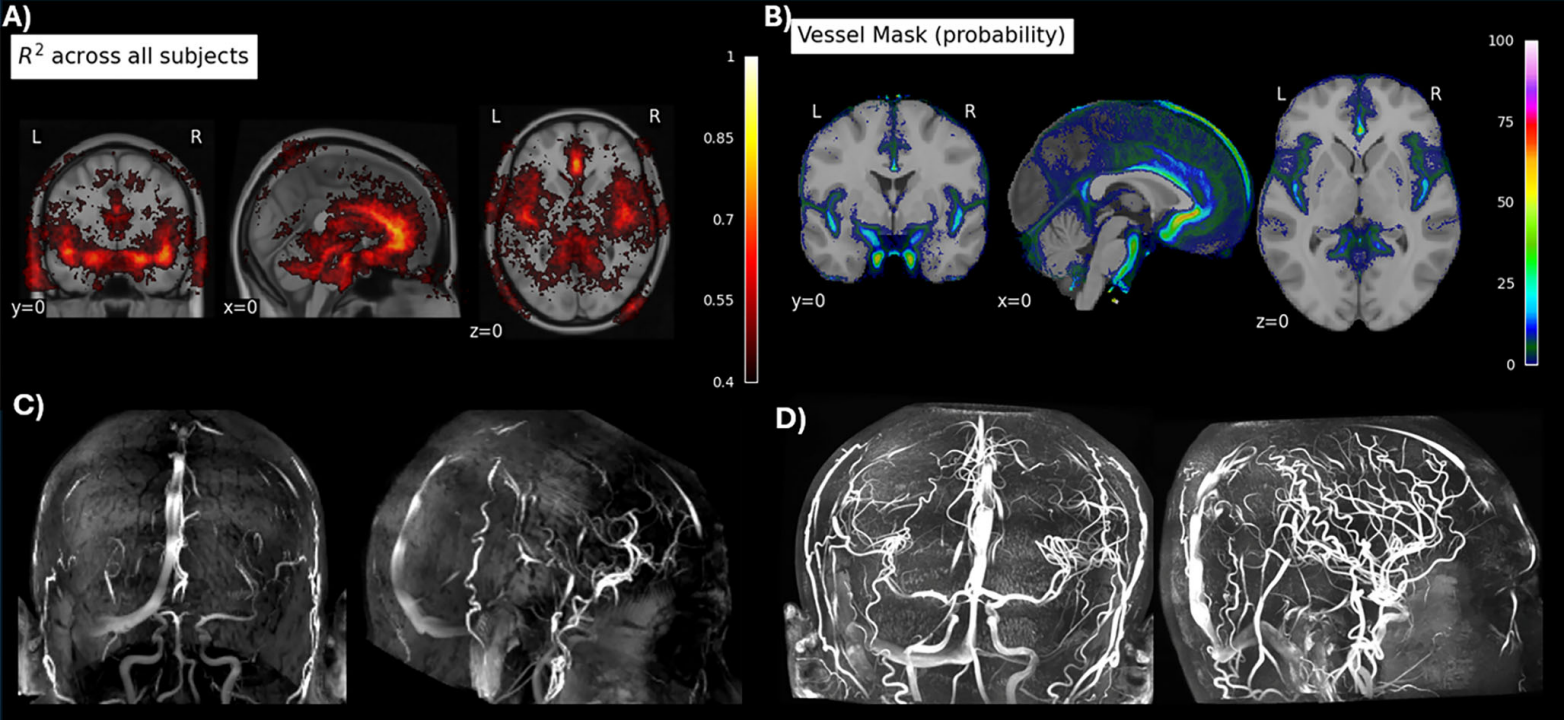
(A) Pulse reliability maps across subjects showing *R* ^2^ > 0.4; (B) visualization of the vessel map atlas from Mouches & Forkert 2019 [52], downsampled to 1 mm isotropic to match the MNI template, then clipped from 0 to 100 to get values corresponding to probability (C) the mean angiogram maximum intensity projection (MIP) across subjects and (D) angiogram MIP for one subject. The angiograms demonstrate the voxels with pulse reliability outside of the brain are still localized to vessels in the head and scalp.

Pulse reliability across all subjects is shown in Figure 3A. The vessel map derived from the atlas from Mouches and Forkert [52] is shown for comparison in Figure 3B. The Spearman correlation coefficients were 0.39 (*p* < 0.0001) for the WM mask and 0.28 (*p* < 0.0001) for the GM mask, showing that the regions with reliable pulsatility were generally localized to the vessels. Graphs of the Spearman correlations between pulse reliability and vessel probability for each voxel are shown in Figure S3. Figure 3C shows the maximum intensity projection (MIP) of the mean angiogram across the subjects and Figure 3D shows an angiogram for a single subject. These angiograms demonstrate that reliable voxels outside of the brain are still localized to vessels in the head and scalp.

### 3.2 Pulsatility

**FIGURE 4:**
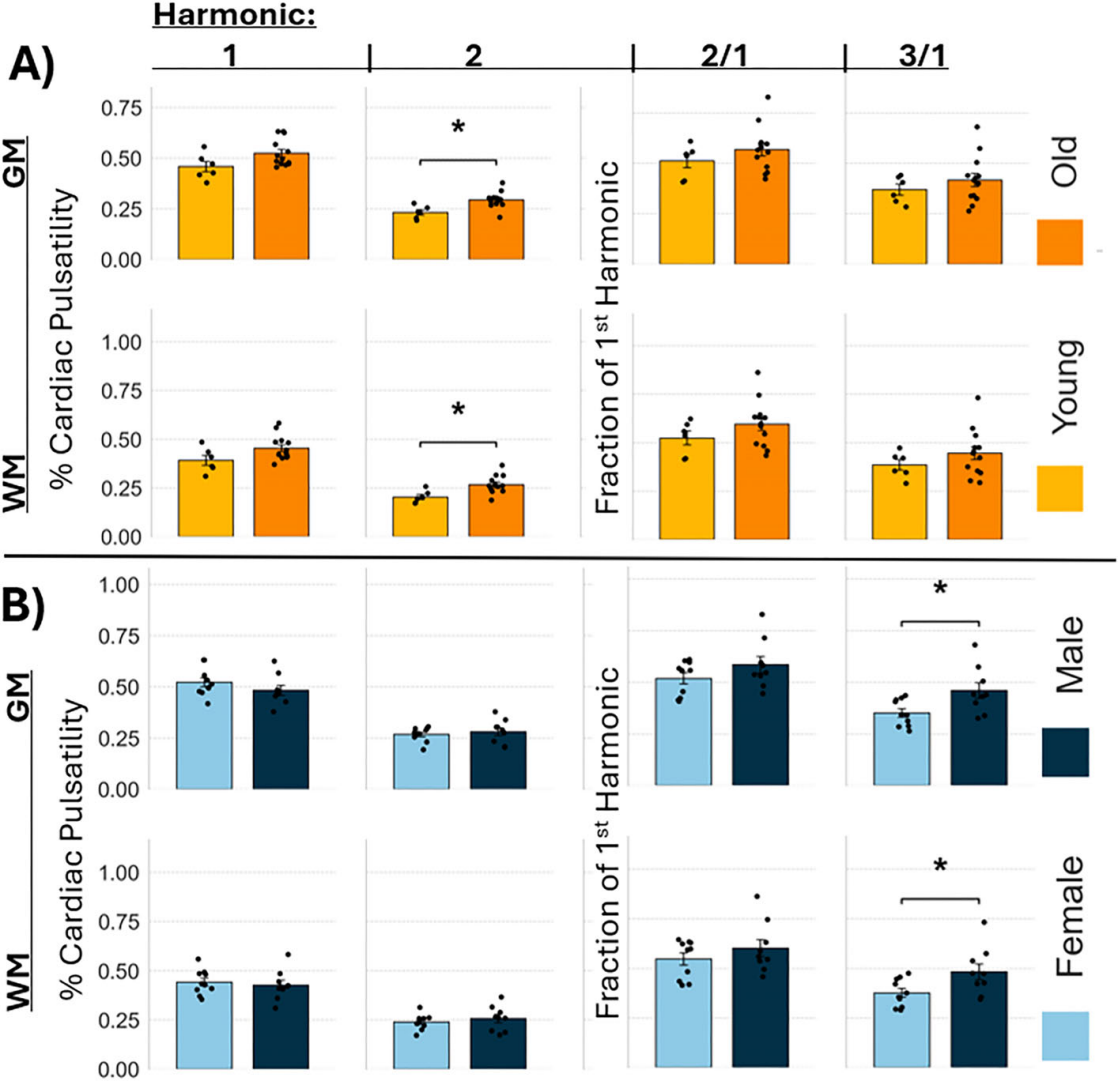
Bar plots representing average % cardiac pulsatility. Group differences are shown for (A) young versus old and (B) male versus female for two harmonics and two harmonic ratios for GM and WM masks. Bars indicate standard errors. Statistical results from OLS with *p* < 0.05 (FDR correction within each column, separately for male/female and young/old) being labeled significant with *.

Examples of pulsatility of the harmonics in individual voxels are shown in Figure 2. A statistical analysis was performed between younger (*N* = 6, aged 25–40 years of age) and older (*N* = 13, aged 55–75 years of age) adults. The results for CoW, CSF, GM, and WM masks, harmonic, and harmonic ratio, with sex used as a covariate, were calculated for the first four harmonics and first three harmonic ratios. Significance (*p* < 0.05 after multiple comparisons correction) was found for GM and WM for the second harmonic, with the first and third harmonics trending toward significance. The older group had a higher magnitude for all significant differences. Plots for each comparison are shown in Figure S4 and summarized for GM and WM masks in Figure 4A for age differences and Figure 4B for sex differences. Males had a larger magnitude in the third harmonic normalized to the first in CoW, CSF, GM, and WM. Cohen's *d*, along with the uncorrected and corrected *p*‐value for each comparison in GM and WM are shown in Tables 2 and 3 for age and sex differences, respectively. Tables S1 and S2 show the full table of values for all masks for age and sex differences, respectively.

**TABLE 2:** Age differences in cardiac pulsatility in GM and WM between young (*n* = 6) and old (*n* = 13) adults (FDR per metric across ROIs).

	Young, *M* ± SE	Old, *M* ± SE	Cohen's *d*	*p*	*q* (FDR)
**GM**					
Harmonic 1	0.458 ± 0.026	0.525 ± 0.019	1.01	0.024*	0.095
Harmonic 2	0.232 ± 0.013	0.294 ± 0.011	1.67	0.006**	0.020*
Harmonic 3	0.166 ± 0.004	0.215 ± 0.012	1.3	0.028*	0.062
Harmonic 4	0.136 ± 0.013	0.158 ± 0.008	0.78	0.171	0.341
Ratio H2/H1	0.513 ± 0.034	0.569 ± 0.031	0.54	0.404	0.538
Ratio H3/H1	0.370 ± 0.027	0.419 ± 0.032	0.46	0.568	0.758
Ratio H4/H1	0.301 ± 0.034	0.305 ± 0.018	0.06	0.913	0.927
**WM**					
Harmonic 1	0.393 ± 0.026	0.455 ± 0.017	1.0	0.048*	0.095
Harmonic 2	0.204 ± 0.013	0.268 ± 0.013	1.54	0.010**	0.020*
Harmonic 3	0.148 ± 0.002	0.200 ± 0.013	1.28	0.031*	0.062
Harmonic 4	0.121 ± 0.012	0.148 ± 0.008	0.93	0.108	0.341
Ratio H2/H1	0.525 ± 0.036	0.597 ± 0.033	0.64	0.284	0.538
Ratio H3/H1	0.386 ± 0.027	0.446 ± 0.034	0.56	0.427	0.758
Ratio H4/H1	0.312 ± 0.033	0.328 ± 0.018	0.22	0.810	0.927

*Note*: The *q*‐value is the *p*‐value after correcting for multiple comparisons.

Abbreviation: Hn, Harmonic *n*.

* Indicates significance at *α* = 0.05.

** Indicates significance at *α* = 0.01.

**TABLE 3:** Sex differences in cardiac pulsatility in GM and WM between female (*n* = 10) and male (*n* = 9) participants (FDR per metric across ROIs).

	Female, *M* ± SE	Male, *M* ± SE	Cohen's *d*	*p*	*q* (FDR)
**GM**					
Harmonic 1	0.522 ± 0.022	0.484 ± 0.024	−0.55	0.089	0.119
Harmonic 2	0.268 ± 0.011	0.281 ± 0.019	0.27	0.915	0.915
Harmonic 3	0.181 ± 0.008	0.220 ± 0.017	0.98	0.073	0.188
Harmonic 4	0.147 ± 0.008	0.156 ± 0.012	0.3	0.710	0.858
Ratio H2/H1	0.520 ± 0.027	0.586 ± 0.040	0.65	0.245	0.349
Ratio H3/H1	0.352 ± 0.020	0.461 ± 0.038	1.2	0.029*	0.038*
Ratio H4/H1	0.285 ± 0.020	0.324 ± 0.023	0.58	0.240	0.321
**WM**					
Harmonic 1	0.442 ± 0.020	0.428 ± 0.025	−0.21	0.380	0.380
Harmonic 2	0.239 ± 0.012	0.257 ± 0.021	0.34	0.763	0.915
Harmonic 3	0.165 ± 0.009	0.205 ± 0.018	0.93	0.094	0.188
Harmonic 4	0.134 ± 0.008	0.146 ± 0.013	0.39	0.588	0.858
Ratio H2/H1	0.548 ± 0.031	0.603 ± 0.043	0.49	0.415	0.415
Ratio H3/H1	0.377 ± 0.022	0.483 ± 0.040	1.09	0.046*	0.046*
Ratio H4/H1	0.306 ± 0.021	0.342 ± 0.024	0.51	0.323	0.323

*Note*: The *q*‐value is the *p*‐value after correcting for multiple comparisons.

Abbreviation: Hn, harmonic *n*.

* Indicates significance at *α* = 0.05.

**FIGURE 5:**
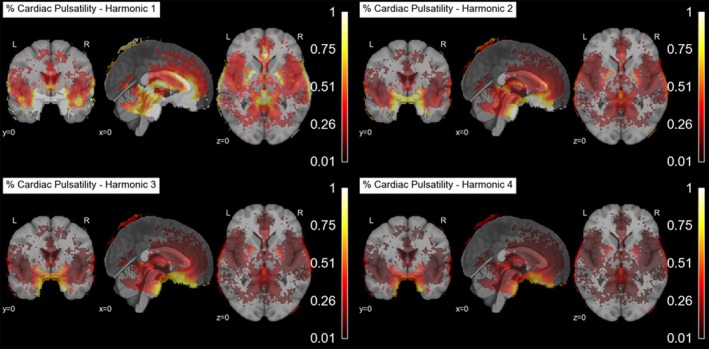
Percent cardiac pulsatility maps for 1st, 2nd, 3rd, and 4th harmonics, clockwise from top left respectively. MNI brain mask and pulse reliability mask *R* ^2^ > 0.4 applied.

The average pulsatility map averaged across all 19 subjects after transforming each subject to MNI is shown in Figure 5 for all harmonics. Percent cardiac pulsatility in the brain was correlated to the pulse reliability as shown by significant rank order Spearman correlations for all harmonics (correlations equal to 0.22, 0.18, 0.13, 0.10 for Harmonics 1, 2, 3, and 4, respectively with *p* < 0.0001 for all harmonics). The first harmonic was correlated to the vessel probability in a mask consisting of voxels in the union of binary WM and GM masks with Spearman correlation of 0.32 (*p* < 0.0001). Plots of the pulse reliability and vessel probability versus percent cardiac pulsatility for each voxel used in the correlation are shown in Figure S5.

### 3.3 Correlation Maps

**FIGURE 6:**
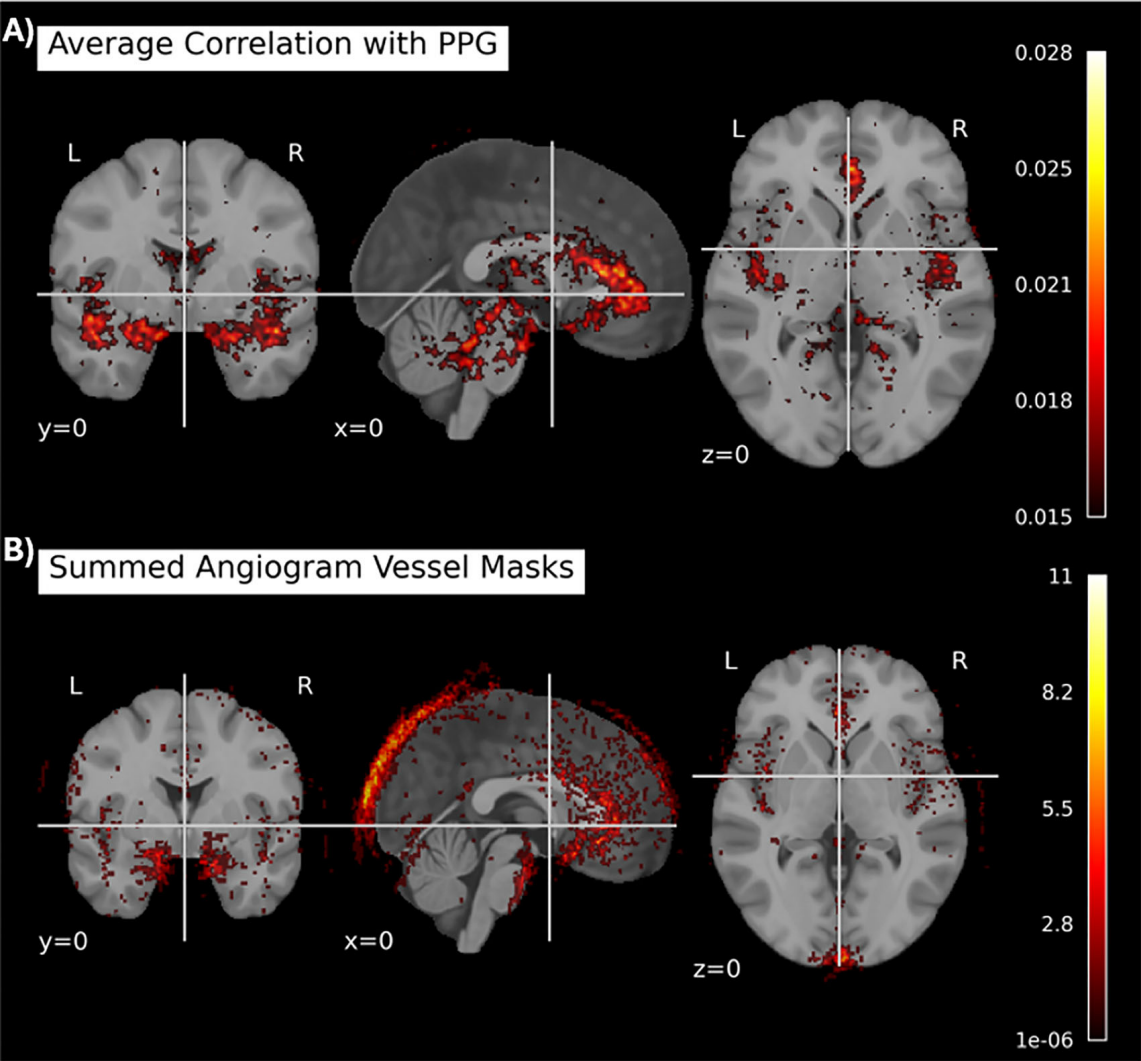
(A) shows magnitude of Pearson correlations for each voxel (normalized) with PPG in MNI space averaged across all subjects thresholded for correlations > 0.015. (B) shows sum of all angiograms masked to vasculature (> 200) transformed to functional and then to MNI space. Taken together these images show overlap between areas with relatively high degree of correlation with pulse and cerebroarterial vasculature as captured by angiography. The highest correlations are primarily clustered near the base of the brain in the area of the circle of Willis (top (A), axial), middle cerebral artery (middle, coronal), and anterior cerebral artery (lower (B), sagittal).

Maps of correlation of the magnitude of pulsations in each voxel with the PPG‐measured pulse are shown in Figure 6. The correlation maps follow the typical vascular structure for major arteries, with higher correlations concentrated near the CoW, bilateral middle cerebral arteries, and anterior cerebral arteries, showing high localization of signal to vascular areas. We note that differential delays between the PPG signal and the pulsation signal in each voxel are expected to impact this correlation; however, significant correlations in vascular regions were observed without compensating for this delay.

## 4 Discussion and Conclusion

The PS‐fMRI sequence achieved 2 mm isotropic, whole brain coverage to enable visualization of pulsation throughout the brain. Odd‐even heartbeat pulse reliability was localized around vascular structures and parenchyma. Pulsatility is correlated with pulse reliability in reliable regions. Using a TR of 65 ms, we were able to resolve the primary cardiac pulsation signal and 4 of its harmonics. Several significant age and sex differences were observed in the pulsation data, in agreement with previous literature. This indicates that the PS‐fMRI sequence is suitable for further work in resolving temporal features of the pulsatile signal throughout the brain.

### 4.1 Age‐ and Sex‐Related Pulsatility

Our results agree with previous literature that shows age‐related increases in pulsatility [9, 21, 24]. Similar to our first harmonic measure, a previous study measured magnitude within 0.02 Hz around the heartbeat frequency and found increases in older adults in normal appearing WM within a limited acquisition of four 5 mm thick slices [24]. Another study defined pulsatility as (maximum of pulse amplitude − minimum of pulse amplitude)/median of a baseline signal within the CoW (single‐slice) and found increased pulsatility within older adults [21], and other studies use phase contrast MRI as opposed to BOLD to detect age‐related increases [9]. Other studies define pulsatility as a metric with combined information from the first three cardiac harmonics, showing that overall the harmonics increase with age [53]. With aging, arterial stiffening is associated with increased amplitude and dominance of the first two harmonics of pulse pressure [54]. The PS‐fMRI cardiac waveform may reflect this by being partially dependent on blood volume. Other factors such as reflected waves, impedance matching at the tissues, blood velocity, and variations in the elasticity of parenchymal tissue may also play a role. The current work furthers previous findings by achieving total brain coverage, fast acquisition, and high spatial resolution. The PS model can be used to expand the ROIs accessible when measuring pulsatility with short TR.

In addition, there are significant sex differences in the CoW, CSF, GM, and WM masks, where males show a larger third harmonic normalized to the first (*p* < 0.05). In the literature, a similar observation was made using tonometry to measure the pulse waveform at the wrist [55], with males having greater third harmonic ratio spectral power [56]. This result may reflect differences in pressure, blood volume, or vascular structure.

The localization of the pulse reliability signal and pulsatility to the vessels, as indicated by the significant correlations, is consistent with previous results [19, 26]. The vessel atlas that was created previously was averaged across subjects, and large arteries are less variable in spatial location than small arteries [52]. For the pulse reliability versus vessel probability, the WM correlation is higher than the GM correlation, indicating the higher the probability of a larger vessel being in a WM voxel. The pulse reliability correlations with all four harmonics show every harmonic considered contributes to pulse reliability. The pulse reliability that we observed in the sagittal sinus is low, while other studies at lower field strength and longer TR show pulse reliability within the sagittal sinus [19]. This may be due to the decrease in T2* at 7 T from the high deoxyhemoglobin in veins.

The source of pulsatility in PS‐fMRI reflects arterial signal decreases during systole seen in Figure 2. Given the 3D sampling of the BOLD signal limiting inflow effects and that these signals are localized to arteries, this signal decrease is likely from a decrease in T2* due to increased blood volume. By the Monro‐Kellie doctrine [57], the CSF volume decreases accompany blood volume increases in the skull, which could cause a signal increase during systole in CSF [19]. The first few harmonics contain the majority of the pulsatile energy in the waveform and the harmonic ratios are related to differences in waveform morphology [32, 33]. Harmonic pulsatility measures would be expected to increase with age, and we see a significant increase in the second harmonic with age. The first harmonic failed to reach significance in this study and future studies with more participants are needed to explore this. In contrast, we expected the harmonic ratios to decrease with age since a filtering effect of the pressure waveform increases with age with more relative energy in the lower harmonics [54, 58]. However, this decrease was not observed in this study.

### 4.2 Limitations

There are several limitations to this study. While the use of a short TR provided the temporal resolution to record over each pulse cycle, shorter TRs also reduce SNR due to the shortened time for longitudinal magnetization recovery [22, 59, 60, 61] and increase the size of the data. The large amount of data (9600 time points of 120 × 120 × 60 voxels per subject) takes a longer time to process and requires a workstation with large memory, as each fMRI data file is > 60 GB.

There are also several limitations with the current sequence. First, whole brain coverage at 65 ms temporal resolution with dynamic imaging requires imaging a spatiotemporal model that leverages redundancy in the image series. With a limited model order, this captures most of the signal in the image series but may lose subtle cardiovascular pulsations that are hard to separate from noise. Further, due to the sampling scheme of serial ordering of kz‐encodings, some spike noise was coherent in our sampling. Although we do not expect cardiac pulsations to be time‐locked to our acquisition, the removal of a small number of spike components (only 160 out of 9600 spectral locations) could result in differential removal of information depending on the specific subject's heart rate.

The analysis was done in voxels with high pulse reliability across subjects, which is related to the larger vessels and their surrounding brain parenchyma. For analysis of smaller arteries or in the presence of vascular pathology, spatial variability may require individual vascular maps. Further, large differences in disease states, such as small vessel disease, can be accompanied by WM lesions where pulsatility is very low due to arterial decay [24]. Other pathologies may affect waveform complexity computed from the cardiac harmonics, such as in Alzheimer's disease [32]. We did not expect large differences in vascular remodeling in the current study as we used healthy old and young adults.

### 4.3 Conclusion

PS‐fMRI is a new fMRI imaging method that uses the PS model to sample whole brain BOLD‐based fMRI, with 2 mm isotropic resolution at an ultra‐fast TR of 65 ms. The pulsatile signal was reliably detected across four harmonics of the heart cycle, localized to the CSF and vessels, and detected significant age and sex differences. This could enable the study of age‐related changes to the elasticity of blood vessels throughout the brain.

## Funding

This work was supported by a seed grant from the Beckman Institute Biomedical Imaging Center. Additional support was provided by the National Institute on Aging, R01AG059878, RF1AG062666. The content is solely the responsibility of the authors and does not necessarily represent the official views of the National Institutes of Health.

## Conflicts of Interest

Authors declare that this study received funding from the Beckman Institute for Advanced Science and Technology at the University of Illinois Urbana Champaign.

## Supporting information


**Table S1:** Age differences in cardiac pulsatility between young ( *n* = 7) and old ( *n* = 14) adults (FDR per metric across ROIs). The *q* ‐value is the *p* ‐value after correcting for multiple comparisons. Hn, harmonic *n* . * indicates significance at *α* = 0.05.
**Table S2:** Sex differences in cardiac pulsatility between female (*n* = 12) and male (*n* = 9) participants (FDR per metric across ROIs). The *q*‐value is the *p*‐value after correcting for multiple comparisons. Hn, harmonic *n*. * indicates significance at *α* = 0.05.
**Figure S1:** (A) Phantom frequency spectrum and filter. After averaging the voxels within the phantom ROI, the magnitude of the FFT of the time series was taken. (B) The filter from masking out spikes above the 1e‐5 (AU) threshold. (C) The first temporal basis function FFT magnitude for one subject and (D) after filtering by multiplication with the filter in B.
**Figure S2:** MNI masks showing how each ROI‐related MNI mask overlapped the pulse reliability with threshold set at *R* ^2^ > 0.4.
**Figure S3:** Hexagonal bins showing density of voxels. (A) Vessel probability versus pulse reliability within *R* ^2^ > 0.05 mask, and vessel probability > 0.05 mask for within the WM > 0.5 mask and (B) within the GM > 0.5 mask, showing the density of voxels in hexagonal bins. *ρ* is the Spearman correlation coefficient and *ρ* is the *p*‐value.
**Figure S4:** Bar plots representing average % cardiac pulsatility. Group differences are shown for (A) young versus old and (B) male versus female for four harmonics and three harmonic ratios. Bars indicate standard errors. Statistical results from OLS with *p* < 0.05 (FDR correction within each column, separately for male/female and young/old) being labeled significant.
**Figure S5:** Hexagonal bins showing density of voxels. (A–D) Pulse reliability versus average magnitude at the harmonic for all four harmonics within the MNI brain mask. (E) Spearman correlation (*ρ*) and *p*‐value (*ρ*) between mean % pulsatility and vessel probability within a mask that is (GM > 0.5 or WM > 0.5).

## Data Availability

Data and code for the current study are available from the corresponding author (B.P.S.) upon reasonable request.
