# Supplementary material for: Ultrafast fMRI Detects Age‐Related Changes in Harmonics of Cardiac Pulsations in the Brain at 7 T
Source: Magn Reson Med. 2026 May 10;96(3):1206–18. doi: 10.1002/mrm.70429 (PMC13327429; doi:10.1002/mrm.70429)
Supplement: Supplementary file 1 — Table S1: Age differences in cardiac pulsatility between young ( n = 7) and old ( n = 14) adults (FDR per metric across ROIs). The q ‐value is the p ‐value after correcting for multiple comparisons. Hn, harmonic n . * indicates significance at α = 0.05. Table S2: Sex differences in cardiac pulsatility between female (n = 12) and male (n = 9) participants (FDR per metric across ROIs). The q‐value is the p‐value after correcting for multiple comparisons. Hn, harmonic n. * indicates significance at α = 0.05. Figure S1: (A) Phantom frequency spectrum and filter. After averaging the voxels within the phantom ROI, the magnitude of the FFT of the time series was taken. (B) The filter from masking out spikes above the 1e‐5 (AU) threshold. (C) The first temporal basis function FFT magnitude for one subject and (D) after filtering by multiplication with the filter in B. Figure S2: MNI masks showing how each ROI‐related MNI mask overlapped the pulse reliability with threshold set at R 2 > 0.4. Figure S3: Hexagonal bins showing density of voxels. (A) Vessel probability versus pulse reliability within R 2 > 0.05 mask, and vessel probability > 0.05 mask for within the WM > 0.5 mask and (B) within the GM > 0.5 mask, showing the density of voxels in hexagonal bins. ρ is the Spearman correlation coefficient and ρ is the p‐value. Figure S4: Bar plots representing average % cardiac pulsatility. Group differences are shown for (A) young versus old and (B) male versus female for four harmonics and three harmonic ratios. Bars indicate standard errors. Statistical results from OLS with p < 0.05 (FDR correction within each column, separately for male/female and young/old) being labeled significant. Figure S5: Hexagonal bins showing density of voxels. (A–D) Pulse reliability versus average magnitude at the harmonic for all four harmonics within the MNI brain mask. (E) Spearman correlation (ρ) and p‐value (ρ) between mean % pulsatility and vessel probability within a mask that is (GM > 0.5 o [file MRM-96-1206-s001.docx]

**Supplementary Information**

**Table S1.** Age differences in cardiac pulsatility between young (*n =* 7) and old (*n =* 14) adults (FDR per metric across ROIs). The q-value is the p-value after correcting for multiple comparisons. Hn = Harmonic *n*. * indicates significance at α = 0.05. ** indicates significance at α = 0.01.

|  | Young  *M* ± *SE* | Old  *M* ± *SE* | Cohen's *d* | *p*-value | *q*-value (FDR) |
| --- | --- | --- | --- | --- | --- |
| CoW | | | | | |
| Harmonic 1 | 1.145 ± 0.093 | 1.326 ± 0.078 | 0.68 | 0.088 | 0.118 |
| Harmonic 2 | 0.572 ± 0.047 | 0.682 ± 0.040 | 0.81 | 0.114 | 0.152 |
| Harmonic 3 | 0.399 ± 0.013 | 0.487 ± 0.035 | 0.82 | 0.17 | 0.227 |
| Harmonic 4 | 0.329 ± 0.030 | 0.359 ± 0.027 | 0.34 | 0.547 | 0.73 |
| Ratio H2/H1 | 0.502 ± 0.025 | 0.520 ± 0.024 | 0.22 | 0.888 | 0.888 |
| Ratio H3/H1 | 0.357 ± 0.024 | 0.377 ± 0.030 | 0.2 | 0.965 | 0.965 |
| Ratio H4/H1 | 0.291 ± 0.029 | 0.275 ± 0.020 | -0.23 | 0.434 | 0.927 |
| CSF | | | | | |
| Harmonic 1 | 0.520 ± 0.044 | 0.506 ± 0.025 | -0.15 | 0.814 | 0.814 |
| Harmonic 2 | 0.252 ± 0.021 | 0.272 ± 0.011 | 0.46 | 0.231 | 0.231 |
| Harmonic 3 | 0.174 ± 0.005 | 0.193 ± 0.010 | 0.59 | 0.322 | 0.322 |
| Harmonic 4 | 0.142 ± 0.016 | 0.141 ± 0.008 | -0.03 | 0.928 | 0.928 |
| Ratio H2/H1 | 0.490 ± 0.030 | 0.548 ± 0.030 | 0.58 | 0.355 | 0.538 |
| Ratio H3/H1 | 0.345 ± 0.028 | 0.392 ± 0.031 | 0.47 | 0.562 | 0.758 |
| Ratio H4/H1 | 0.278 ± 0.031 | 0.283 ± 0.017 | 0.08 | 0.927 | 0.927 |
| GM | | | | | |
| Harmonic 1 | 0.458 ± 0.026 | 0.525 ± 0.019 | 1.01 | 0.024* | 0.095 |
| Harmonic 2 | 0.232 ± 0.013 | 0.294 ± 0.011 | 1.67 | 0.006** | 0.020* |
| Harmonic 3 | 0.166 ± 0.004 | 0.215 ± 0.012 | 1.3 | 0.028* | 0.062 |
| Harmonic 4 | 0.136 ± 0.013 | 0.158 ± 0.008 | 0.78 | 0.171 | 0.341 |
| Ratio H2/H1 | 0.513 ± 0.034 | 0.569 ± 0.031 | 0.54 | 0.404 | 0.538 |
| Ratio H3/H1 | 0.370 ± 0.027 | 0.419 ± 0.032 | 0.46 | 0.568 | 0.758 |
| Ratio H4/H1 | 0.301 ± 0.034 | 0.305 ± 0.018 | 0.06 | 0.913 | 0.927 |
| WM | | | | | |
| Harmonic 1 | 0.393 ± 0.026 | 0.455 ± 0.017 | 1.0 | 0.048* | 0.095 |
| Harmonic 2 | 0.204 ± 0.013 | 0.268 ± 0.013 | 1.54 | 0.010** | 0.020* |
| Harmonic 3 | 0.148 ± 0.002 | 0.200 ± 0.013 | 1.28 | 0.031* | 0.062 |
| Harmonic 4 | 0.121 ± 0.012 | 0.148 ± 0.008 | 0.93 | 0.108 | 0.341 |
| Ratio H2/H1 | 0.525 ± 0.036 | 0.597 ± 0.033 | 0.64 | 0.284 | 0.538 |
| Ratio H3/H1 | 0.386 ± 0.027 | 0.446 ± 0.034 | 0.56 | 0.427 | 0.758 |
| Ratio H4/H1 | 0.312 ± 0.033 | 0.328 ± 0.018 | 0.22 | 0.810 | 0.927 |

**Table S2.** Sex differences in cardiac pulsatility between female (*n =* 12) and male (*n =* 9) participants (FDR per metric across ROIs). The q-value is the p-value after correcting for multiple comparisons. Hn = Harmonic *n*. * indicates significance at α = 0.05. ** indicates significance at α = 0.01.

|  | Female  *M* ± *SE* | Male  *M* ± *SE* | Cohen's *d* | *p*-value | *q*-value (FDR) |
| --- | --- | --- | --- | --- | --- |
| CoW | | | | | |
| Harmonic 1 | 1.355 ± 0.098 | 1.172 ± 0.066 | -0.69 | 0.072 | 0.119 |
| Harmonic 2 | 0.652 ± 0.043 | 0.642 ± 0.052 | -0.07 | 0.637 | 0.915 |
| Harmonic 3 | 0.427 ± 0.023 | 0.496 ± 0.047 | 0.63 | 0.282 | 0.376 |
| Harmonic 4 | 0.343 ± 0.024 | 0.357 ± 0.036 | 0.14 | 0.858 | 0.858 |
| Ratio H2/H1 | 0.485 ± 0.018 | 0.547 ± 0.029 | 0.84 | 0.107 | 0.349 |
| Ratio H3/H1 | 0.321 ± 0.015 | 0.425 ± 0.036 | 1.3 | 0.016* | 0.038* |
| Ratio H4/H1 | 0.257 ± 0.015 | 0.306 ± 0.028 | 0.72 | 0.112 | 0.321 |
| CSF | | | | | |
| Harmonic 1 | 0.564 ± 0.028 | 0.451 ± 0.020 | -1.5 | 0.006** | 0.024* |
| Harmonic 2 | 0.278 ± 0.011 | 0.252 ± 0.017 | -0.59 | 0.149 | 0.596 |
| Harmonic 3 | 0.181 ± 0.006 | 0.193 ± 0.014 | 0.39 | 0.535 | 0.535 |
| Harmonic 4 | 0.147 ± 0.010 | 0.136 ± 0.010 | -0.36 | 0.457 | 0.858 |
| Ratio H2/H1 | 0.501 ± 0.026 | 0.563 ± 0.038 | 0.63 | 0.261 | 0.349 |
| Ratio H3/H1 | 0.328 ± 0.020 | 0.433 ± 0.036 | 1.2 | 0.029* | 0.038* |
| Ratio H4/H1 | 0.263 ± 0.018 | 0.302 ± 0.022 | 0.63 | 0.204 | 0.321 |
| GM | | | | | |
| Harmonic 1 | 0.522 ± 0.022 | 0.484 ± 0.024 | -0.55 | 0.089 | 0.119 |
| Harmonic 2 | 0.268 ± 0.011 | 0.281 ± 0.019 | 0.27 | 0.915 | 0.915 |
| Harmonic 3 | 0.181 ± 0.008 | 0.220 ± 0.017 | 0.98 | 0.073 | 0.188 |
| Harmonic 4 | 0.147 ± 0.008 | 0.156 ± 0.012 | 0.3 | 0.710 | 0.858 |
| Ratio H2/H1 | 0.520 ± 0.027 | 0.586 ± 0.040 | 0.65 | 0.245 | 0.349 |
| Ratio H3/H1 | 0.352 ± 0.020 | 0.461 ± 0.038 | 1.2 | 0.029* | 0.038* |
| Ratio H4/H1 | 0.285 ± 0.020 | 0.324 ± 0.023 | 0.58 | 0.240 | 0.321 |
| WM | | | | | |
| Harmonic 1 | 0.442 ± 0.020 | 0.428 ± 0.025 | -0.21 | 0.380 | 0.380 |
| Harmonic 2 | 0.239 ± 0.012 | 0.257 ± 0.021 | 0.34 | 0.763 | 0.915 |
| Harmonic 3 | 0.165 ± 0.009 | 0.205 ± 0.018 | 0.93 | 0.094 | 0.188 |
| Harmonic 4 | 0.134 ± 0.008 | 0.146 ± 0.013 | 0.39 | 0.588 | 0.858 |
| Ratio H2/H1 | 0.548 ± 0.031 | 0.603 ± 0.043 | 0.49 | 0.415 | 0.415 |
| Ratio H3/H1 | 0.377 ± 0.022 | 0.483 ± 0.040 | 1.09 | 0.046* | 0.046* |
| Ratio H4/H1 | 0.306 ± 0.021 | 0.342 ± 0.024 | 0.51 | 0.323 | 0.323 |


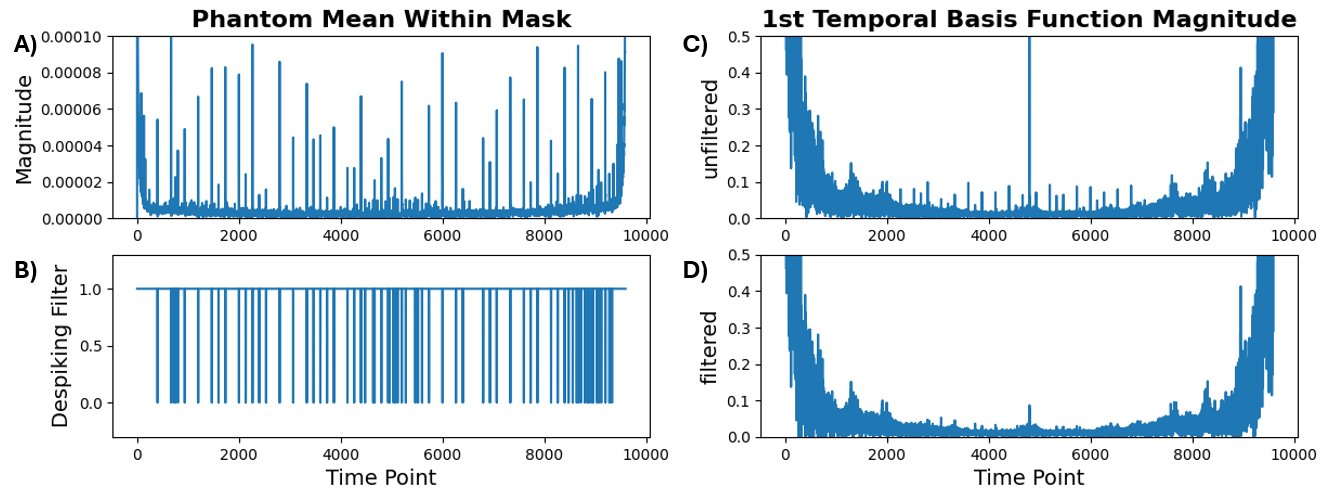
**Supplementary Figure S1.** A) Phantom frequency spectrum and filter. After averaging the voxels within the phantom ROI, the magnitude of the FFT of the time series was taken. B) The filter from masking out spikes above the 1e-5 (AU) threshold. C) The first temporal basis function FFT magnitude for one subject and D) after filtering by multiplication with the filter in B.


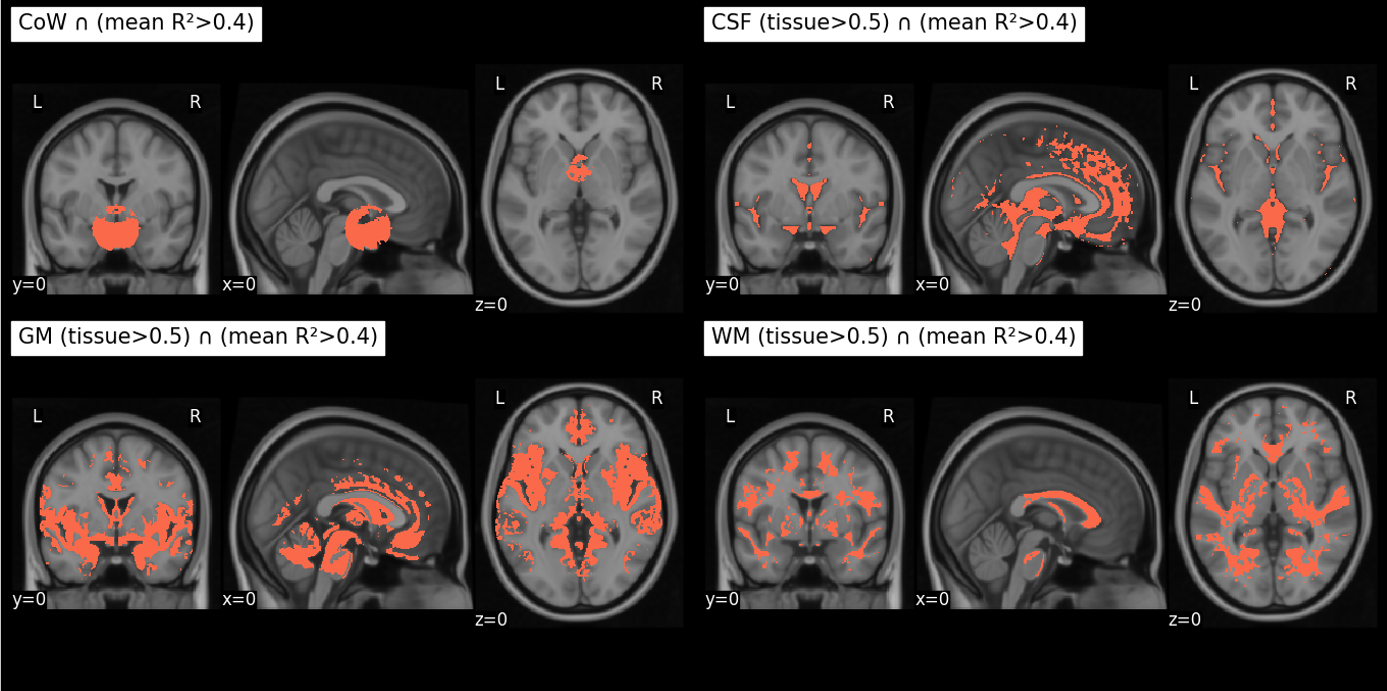


**Supplementary Figure S2:** MNI masks showing how each ROI-related MNI mask overlapped the pulse reliability with threshold set at $R^{2}>0.4$.


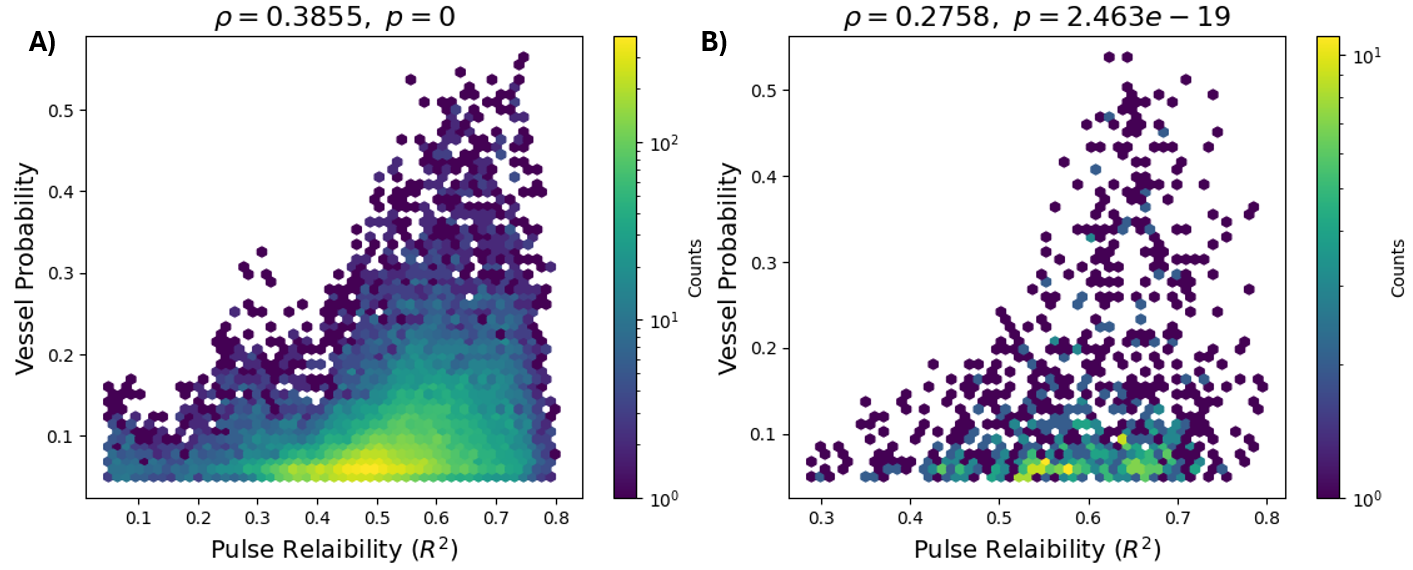
**Supplementary Figure S3.** Hexagonal bins showing density of voxels. A) Vessel probability vs pulse reliability within R^2^ > 0.05 mask, and vessel probability > 0.05 mask for within the WM > 0.5 mask and B) within the GM > 0.5 mask, showing the density of voxels in hexagonal bins. 𝜌 is the Spearman correlation coefficient and 𝑝 is the p-value.


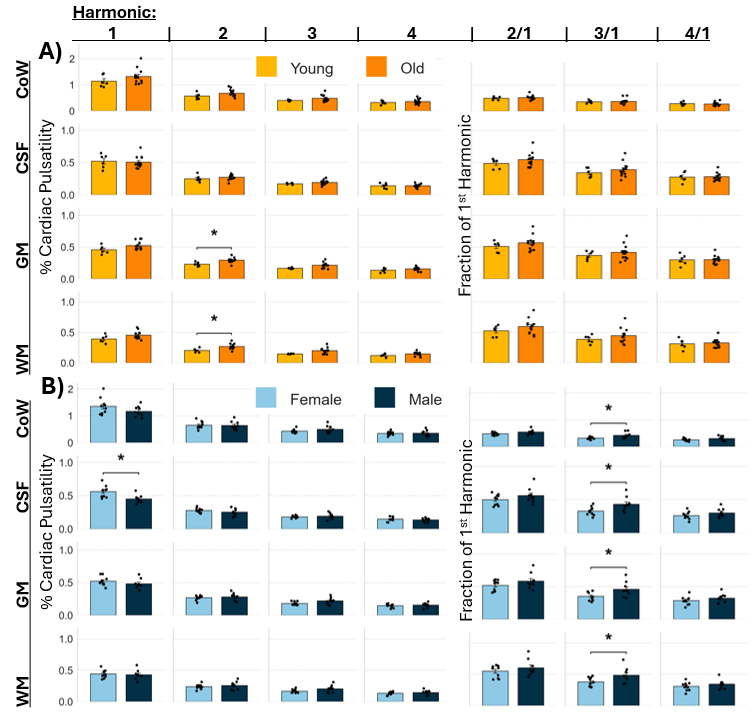


**Supplementary Figure S4.** Bar plots representing average % cardiac pulsatility. Group differences are shown for a) young vs old and b) male vs female for 4 harmonics and 3 harmonic ratios. Bars indicate standard errors. Statistical results from OLS with p < 0.05 (FDR correction within each column, separately for male/female and young/old) being labeled significant with *.


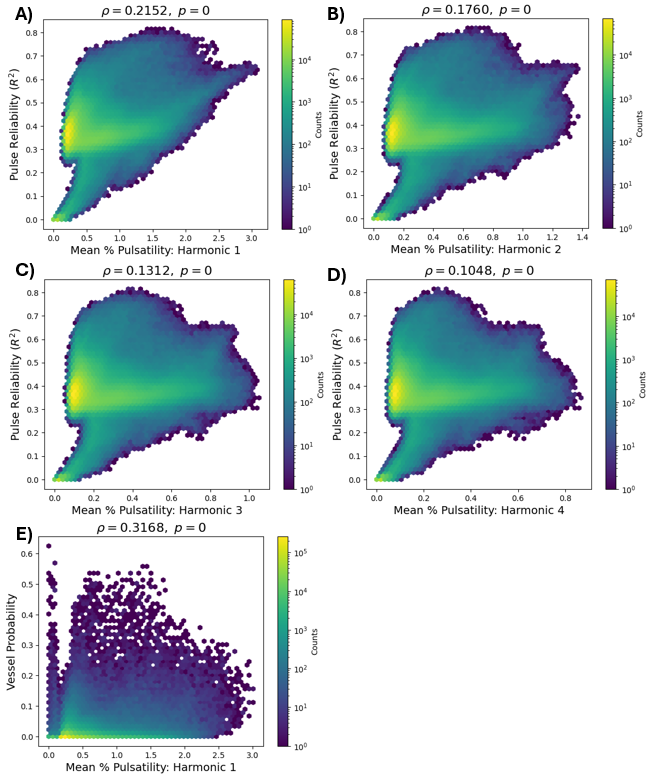


**Supplementary Figure S5.** Hexagonal bins showing density of voxels. A-D) Pulse reliability vs average magnitude at the harmonic for all 4 harmonics within the MNI brain mask. E) Spearman correlation (𝜌) and p-value (𝑝) between mean % pulsatility and vessel probability within a mask that is (GM > 0.5 or WM > 0.5).
